# Cardiovascular Effects of Shock and Trauma in Experimental Models. A Review

**DOI:** 10.5935/1678-9741.20150065

**Published:** 2016

**Authors:** Mauricio Rocha-e-Silva

**Affiliations:** 1 Faculdade de Medicina da Universidade de São Paulo (FMUSP), São Paulo, SP, Brazil.

**Keywords:** Cardiovascular Diseases, Cardiovascular System, Microcirculation, Myocardial ischemia, Shock, Animal Models

## Abstract

Experimental models of human pathology are useful guides to new approaches towards improving clinical and surgical treatments. A systematic search through PubMed using the syntax (shock) AND (trauma) AND (animal model) AND (cardiovascular) AND ("2010/01/01"[PDat]: "2015/12/31"[PDat]) found 88 articles, which were reduced by manual inspection to 43 entries. These were divided into themes and each theme is subsequently narrated and discussed conjointly. Taken together, these articles indicate that valuable information has been developed over the past 5 years concerning endothelial stability, mesenteric lymph, vascular reactivity, traumatic injuries, burn and sepsis. A surviving interest in hypertonic saline resuscitation still exists.

## INTRODUCTION

It is a truth universally acknowledged that experimental models of human pathology are useful guides to new approaches towards improving clinical and surgical treatments, even though transposition is rarely perfect and often unacceptable. It is the author's experience after a long life in this field of research that many of the procedures routinely used in the evaluation and treatment of shock stem from work initially performed in the framework of carefully experiments. This review intends to cover reports from experimental models relating to the effects of shock and trauma upon the cardiovascular system published during a five-year period which ended on December 31, 2014. I have found that data published during these five years cover some classic areas of investigation (e.g., hemorrhagic shock, aortic occlusion, trauma, traumatic brain injury and sepsis), but also a number of new lines, such as endothelial integrity and vascular reactivity. The concept behind this critical review is to examine these contributions and the manner in which they contribute to our contemporary understanding of shock. I was also glad to note that hypertonic resuscitation, to which I contributed during my active research life, is still a subject of interest.

## METHODS

Articles were selected from pubmed.gov using the following search syntax: (shock) AND (trauma) AND (animal model) AND (cardiovascular) AND ("2010/01/01"[PDat]: "2015/12/31"[PDat]). The search yielded 88 articles; the abstracts were subsequently examined manually, leading to a final collection of 55 entries. A careful examination of the texts further reduced this number to 43; four other articles^[1-4]^ published previously to the time window covered in this review period are cited in order to clarify items discussed herein^[3]^. The 43 contemporary articles were then divided into themes and each theme is subsequently narrated and discussed conjointly.

## RESULTS AND DISCUSSION

Classic physiological characteristics of shock were studied in four reports. Jonker et al.^[5]^ examined the impact of hypovolemic shock on the aortic diameter in a porcine model to determine its implications for the endovascular management of hypovolemic patients with traumatic thoracic aortic injury. The aortic diameter was significantly decreased during blood loss. This very basic anatomical/physiological study may have implications for thoracic endovascular aortic repair because endografts should either be made oversize, or additional imaging after fluid resuscitation may be required for the production of an adequate graft. Transcapillary refill was evaluated in dogs submitted to uncontrolled hemorrhage by Sallum et al.^[6]^ I am a co-author of this paper and we found that rebleeding occurred irrespective of whether the condition was treated with isotonic or hypertonic saline. However, as might be expected from some strictly physical considerations, hypertonic saline treatment induced transcapillary refill whereas isotonic saline resuscitation led to capillary extravasation. This was one piece of research undertaken at the Heart Institute Research Division in response to claims that hypertonic saline resuscitation should be avoided due to its capacity to increase bleeding^[3]^. Chao et al.^[7]^ studied temporary vascular shunts, because the procedure is frequently relied upon in current military scenarios: pigs were submitted to 40% of blood loss and the study aimed at establishing the time to failure of short proximally placed vascular shunts and to examine histological changes in shunted arteries. These shunts remained functional for at least 48-72 hours. This piece of information may aid in the establishment of combat and mass disaster evacuation patterns. Pasupuleti et al.^[8]^ examined the effects of beta-1 blockers in a rat model of lung contusion and hemorrhagic shock; they claim that its use was ineffective in protecting the bone marrow cellularity. Even though this is a negative result and I feel that its presence here is justified in order to prevent would-be researchers from repeating it.

### Hemorrhage

The most frequently reported studies refer to animal models in which a single hemorrhagic stroke was imposed upon the experimental model.

### Endothelial integrity and protection

The endothelium is the first line of defense of end-organs against external aggression. Research around this theme indicates that the deleterious effects of hemorrhagic shock can be prevented through endothelial protection. Causey et al.^[9]^ reports on data obtained from experiments in swine, in which a supraceliac cross clamp was applied simultaneously with a hemorrhagic stroke; valproic acid was administered simultaneously. Valproic acid reduced resuscitative requirements, and led to improved hemodynamics; evaluation of gene expression for specific growth factors showed that the treatment also minimized pathologic endothelial cell dysfunction through the expression of genes regulating endothelial transforming growth factors. They conclude that these genes were involved in the preservation of endothelial function, which was enhanced by valproic acid. In a second study^[10]^, using a similar model, Causey et al.^[9]^ formulated a concept that valproic acid confers relevant metabolic, cardiovascular and pathologic protection to individuals submitted to hemorrhagic hypotension. Still on the theme of valproic acid, Dekker et al.^[11]^ used a swine model in which traumatic brain injury combined with hemorrhage was treated with fresh frozen plasma and valproic acid. In this clinically relevant large animal model of combined brain trauma and hemorrhage, the addition of valproic acid to fresh frozen plasma resulted in an early upregulation of platelet activation in the circulation and in the brain. I definitely believe that this is a theme that encourages future research. Endothelial integrity is the object of a study by Torres et al.^[12]^, who evaluated the effects of hetastarch, lactated Ringer's or fresh frozen plasma on endothelial glycocalyx, venular blood flow and coagulation in hemorrhaged rats. I believe it is useful to interrupt the narrative of the Torres et al.^[12]^ findings with a note on glycocalyx: this is a glycoprotein-polysaccharide covering endothelial cell membranes which acts as an identifier capable of distinguishing between its own healthy cells, transplanted tissues, diseased cells, or invading organisms. Torres et al.^[12]^ claim that their findings support the concept of cardiovascular and microvascular stabilization by infused fresh frozen plasma, in which the increase in microvascular perfusion associated with restored endothelial glycocalyx is essential for an optimal resuscitation strategy. The concept of glycocalyx integrity is further discussed in a situation of traumatic brain injury. Using a rat model, Jepsen et al.^[13]^ examined the hypothesis that severe isolated traumatic brain injury causes shedding of endothelial glycocalyx as a consequence of the injury, and that this shedding is enhanced by valproic acid, which diminished the size of the brain lesion caused by the injury. Thus, rather paradoxically, it appears that the shedding of glycocalyx may be beneficial to the response of the brain to trauma. More research is clearly required to resolve these antagonistic reports. The role of endothelial integrity is also the object of a study by Johnson et al.^[14]^, who report that, in a rat model of severe hemorrhage, arginase, an enzyme that competes for l-arginine with nitric oxide synthase, plays an essential role in the development of endothelial dysfunction in resistance vessels. In contrast, the ministration of exogenous nitric oxide to hamsters submitted to hemorrhagic shock by Nachuraju et al.^[15]^ preserved microvascular perfusion and reduced hemorrhagic shock sequelae. The relevance, stability, and efficacy of exogenous, NO therapy in the form of NO-nanoparticles will potentially facilitate its intended use in trauma situations. Pati et al.^[16]^ showed that the protective effects of fresh frozen plasma on endothelial permeability after hemorrhagic shock is time dependent and diminishes from day 1 to day 5 after the plasma is thawed. Deitch et al.^[17]^ tested the hypothesis that trauma and hemorrhage are capable of inducing erythrocyte-endothelial adhesion. They show that erythrocytes obtained from rats subjected to trauma-hemorrhage can be used to determine the role of endothelial receptors in the increased adhesive response. It is known that red blood cells normally express the CD36 adhesion molecule, which normally binds to endothelial cell receptors. In conditions of trauma-hemorrhage mesenteric lymph carries gut derived factors, which induce enhanced CD36 expression. These findings may explain the microvascular dysfunction occurring after trauma-hemorrhagic shock, sepsis, and other stress states.

### Vascular reactivity

Vascular reactivity is an obviously significant element in the natural history of shock and has been frequently examined during this period. Zhu et al.^[18]^ describe the role of adenosine A2A receptors in organ specific vascular reactivity following hemorrhagic shock in rats. They studied the thoracic aorta, and samples of femoral, renal, superior mesenteric, pulmonary and middle cerebral arteries. Interestingly, the reactivity of the femoral, mesenteric, renal and middle cerebral arteries increased during early shock and decreased thereafter, whereas that of aorta end pulmonary artery decreased throughout the period. The interesting point is that the rate of loss of vascular reactivity in the different vessels was negatively correlated with adenosine A2A receptors expression levels in normal and shock conditions. They conclude that the expression of these receptors may have beneficial effects on shock by improving vascular reactivity and hemodynamic parameters. The problem of vascular reactivity was also studied by Yang et al.^[19]^, who claim that mitogenactivated protein kinases are essential for the regulation of mesenteric arteries following shock. 

Xu et al.^[20]^ have likewise connected protein Kinase C to hemorrhagic shock; they reported experiments performed in superior mesenteric arteries of rats which show that the kinase is involved in the regulation of calcium sensitivity after hemorrhagic shock; the use of antagonists of this signaling molecule decrease and aggravate calcium desensitization induced by shock. In the same line, Hu et al.^[21]^ report on the effect of the BK Ca^++^ activated potassium channel activator NS1619 against shock induced vascular hyporeactivity. They found that if rats were pretreated with the channel molecule, then submitted to hemorrhagic shock, their 72 hours survival rate was increased and suggest the BK Ca^++^ channel may be a target for the treatment of shock induced vascular hyporeactivity. This is probably a line of research that may add essential tools for the treatment of the more pernicious shock conditions.

### Mesenteric lymph and shock

Shock has been long recognized as an essential factor in the breakdown of the gut-blood defense barrier. It would thus be obvious to expect that mesenteric lymph might be an essential culprit in the development of organ dysfunction in this condition. In a previous study, Sambol et al.^[2]^ had noted that the myocardial dysfunction which follows trauma and/or hemorrhage can be prevented by mesenteric lymph duct ligation; in their present study^[22]^ they show that mesenteric lymph collected from rats submitted to trauma plus hemorrhage and applied to isolated cardiac myocytes induced an immediate increase in the amplitude of cell shortening followed by a complete block of contraction, concomitant with a similar dual positive - negative effect on Ca^++^ transients. It thus appears that T/HS lymph directly causes negative inotropic effects on the myocardium and that lymph from trauma/hemorrhaged rats induced changes in myocyte function that are likely to contribute to the development of myocardial dysfunction. I see this as a classic type of evidence that was first developed as the postulate on chemical mediation proposed in 1934 by Nobel laureate Sir Henry Dale^[4]^. Morishita et al.^[23]^ studied the effects of post-hemorrhagic shock mesenteric lymph collected from rats on isolated human neutrophils and conclude that this lymph contains biologically active lipids, which may be involved in the pathogenesis of acute lung injury and/or multiple-organ dysfunction syndrome. More recently, Zhao et al.^[24]^ induced shock in rats by superior mesenteric artery occlusion and studied the effect of mesenteric lymph reperfusion. The mesenteric lymph duct was occluded for one hour. The occlusion induced increased levels of the endotoxin-lipopolysaccharide receptor, of the lipopolysaccharide-binding protein, of the intercellular adhesion molecule-1 and of tumor necrosis factor-α. Concurrently, mesenteric lymph reperfusion after superior mesenteric artery occlusion further aggravated these deleterious effects. Thus, lymph reperfusion exacerbated the endotoxin translocation in brain; thereby an increased inflammatory response occurred, suggesting that the intestinal lymph pathway plays an important role in the brain injury after superior mesenteric artery occlusion shock.

### Aortic occlusion

Two papers analyzed the potential therapeutic effect of aortic occlusion after severe blood loss. Avaro et al.^[25]^ described the feasibility of aortic balloon catheter occlusion in intra-abdominal hemorrhage in pigs and found that the occlusion, followed by surgical damage control improved survival in this animal model of uncontrolled hemorrhagic shock caused by abdominal trauma. Thus, aortic balloon occlusion could be considered for the management of severe abdominal trauma. Along the same line, Markov et al.^[26]^ employed a porcine model to test the effects of resuscitative endovascular balloon occlusion of the aorta. Their results indicate that shock improves mean central aortic pressure, but is associated with a greater lactate burden; however, this lactate burden returned to control levels within the study period. They claim that the procedure is ultimately survivable and potentially life-saving. This is still a very precarious concept requiring further examination before being even considered for clinical use. Rocha et al.^[27]^ analyzed pulse pressure variation in anesthetized male pigs under four different respiratory regimens: I) normovolemia and spontaneous breathing; II) hypovolemia and spontaneous breathing; III) hypovolemia under mechanical ventilation; and IV) after volume replacement, under mechanical ventilation. Cardiac output, pulmonary artery occlusion pressure, systolic pressure variation, mean arterial pressure, and heart rate were measured at all stages; red blood cell count was determined at stages I, II, and IV. Pulse pressure variation values were higher during spontaneous breathing than during mechanical ventilation. This may be a useful model for the assessment of fluid volume, with baseline values as a starting point to which serial measurements should be compared after the institution of specific therapies.

### Trauma

A number of animal trials have been performed on the combined effects of hemorrhage and trauma. Here we deal only with trauma not affecting the brain, which will be the object of the next section. Nöt et al.^[28]^ studied the effects of glucosamine (to increase O-GlcNAc synthesis) or O-(2-acetamido-2-deoxy-D-glucopyranosylidene) amino N-phenyl carbamate - PUGNAc (to inhibit O-GlcNAc removal) in rats subjected to a trauma-hemorrhage protocol. By way of clarifying concepts I have allowed myself to note that O-GlcNAc is a special type of protein glycosylation factor that acts exclusively within the nuclear and cytoplasmic compartments of the cell. The maintenance of O-GlcNAc synthesis (through glucosamine or PUGNAC) was shown to increase survival of hemorrhage/traumatized rats. However, only PUGNAc treatment attenuated significantly the subsequent tissue injury and inflammatory responses, suggesting that inhibition of O-GlcNAc removal may represent a new therapeutic approach for the treatment of hypovolemic shock. Kim et al.^[29]^ evaluated the effect of 17β-estradiol administration on cardiovascular parameters in male rats after trauma-hemorrhage for an extended period (3 hours) of severe hypotension; this study was based on single photon emission computed tomography imaging performed on the blood-pool. They found a significantly larger blood volume in heart, kidney, and liver of rats after therapy, which supports the notion that 17β-estradiol produces salutary effects on the cardiovascular system after trauma-hemorrhage. Chai et al.^[30]^ studied the effect of exogenous hydrogen sodium sulfide (NaHS) on the evolution of trauma + hemorrhage in rats resuscitated with Ringer lactate, with/without the addition of hydrogen sulfide. NaHS resulted in an increase in mean arterial blood pressure, positive and negative left ventricular pressure [dP/dt(max)]. Aspartate aminotransferase, alanine aminotransferase, blood urea nitrogen, and serum creatinine were reduced in the NaHS-treated group. NaHS also significantly reduced the mortality rate at 24h. The study demonstrates that exogenous NaHS confers protective effects after trauma-hemorrhage and resuscitation, by preventing a decrease in the antioxidant defense system. Once again, it is a study that requires more research but such research is certainly justified by results so far expounded.

Resveratrol has been shown to have protective effects for patients in shock-like states, and protein kinase B is known to play a role in pro-inflammatory events in response to injury. Tsai et al.^[31]^ endeavored to determine whether resveratrol provides cardio-protection mediated via an protein kinase B -dependent pathway in rats submitted to trauma plus hemorrhage. Their results suggest that resveratrol attenuates cardiac injury following trauma-hemorrhage, which is, at least in part, due to its anti-inflammatory effects via the protein kinase B - dependent pathway. Pulathan et al.^[32]^ evaluated the effect of ethyl pyruvate on the systemic inflammatory response and lung injury in an experimental rat model of ruptured abdominal aortic aneurysm. Compared to untreated animals, rats treated with ethylpyruvate presented reduced levels of serum myeloperoxydase, malondialdehyde, and tumor necrosis factor-a. They conclude that ethyl pyruvate reduces systemic inflammatory response and lung injury resulting from trauma and shock. Taghavi et al.^[33]^ note that patients suffering from penetrating trauma and severe hemorrhagic shock are at risk of receiving positive pressure ventilation during transport to definitive care. They claim that this mode of ventilation has harmful physiologic effects in severe low-flow states and that a different regimem, termed permissive hypoventilation would result in better outcomes. Therefore they set up a swine model to test the concept that "permissive hypoventilation," where manual breaths are not given and oxygen is administrated passively via facemask. They found that although permissive hypoventilation leads to respiratory acidosis, it results in less hemodynamic suppression and better perfusion of vital organs. They suggest that in severely injured patients, with penetrating trauma, consideration should be given to immediate transportation, rather than to positive pressure breathing procedures. This is part of a rather controversial concept according to which rapid transport to an emergency care unit is more essential than pre-hospital interventions. It is quite obvious that trauma, in itself defies concepts of simple unilateral solutions. The same will apply to the reports under the next three headings, namely traumatic brain injury, burn and sepsis.

### Traumatic brain injury

Seven papers analyzed animal models of traumatic brain injury. The lingering controversy on whether hypertonic saline or mannitol is more effective in the treatment of traumatic brain injury was revisited by Marks et al.^[34]^ they find that the two formulae are undistinguishable in terms of the activation of the neutrophil-endothelial interaction in the brain. However, additional studies may be needed to determine whether either type of osmotherapy has more subtle effects on the interaction in terms of a potential clinical advantage. Chen et al.^[35]^ note that traumatic brain injury may influence the physiological response to acute blood loss and endeavored to determine if the combination of severe hemorrhage and standardized brain trauma may influence the dynamics of the microcirculation. This study was performed in rats; the effects of hemorrhage vs. traumatic brain injury upon red blood cell velocity and blood flow in arterioles and venules were compared. Venular dynamics was similar in both groups, arteriolar velocity and blood flow decreased to a similar extent in both groups; however, 60 min after the insult an overshoot of velocity and flow was observed exclusively in the combined insult group. It thus appears that the brain insult modulated arteriolar responses to hemorrhage in third-order vessels, but further research is necessary to precisely define the role of brain insult on the microcirculation in tissues vulnerable to hemorrhage. Skotak et al.^[36]^ describe a new model of graded neurotrauma by varying the helium pressure in a trauma tube applied to rats. The immunostaining for immunoglobulin G of brain sections of these rats sacrificed 24 hours afer exposure showed a diffuse breakdown of the blood-brain barrier in the cerebral parenchyma. A non-linear function of acute response and of mortality vs. peak helium pressure is reported. Abdul-Muneer et al.^[37]^ induced mild traumatic head injury in rats and found that brain injury is initiated by the induction of the free radical-generating enzymes NADPH oxidase-1 and inducible nitric oxide synthase. This hypothesis was further confirmed by the activation of caspase-3 and cell apoptosis mostly around the perivascular region. As discussed earlier, Jepsen et al.^[13]^ examined the hypothesis that severe isolated traumatic brain injury causes shedding of endothelial glycocalyx. Also as previously noted, the interaction of valproic acid with traumatic brain injury was reported by Dekker et al.^[11]^ in a clinically relevant large animal model of combined brain trauma and hemorrhage. Dekker et al.^[38]^ examined the interaction of traumatic brain injury and hemorrhage in swine treated with normal saline, hypertonic saline + hetastarch or fresh frozen plasma. The main object of this investigation was to determine the effects of the alternative treatments on coagulation and endothelial function. Normal saline was associated with an early activation of coagulation, anticoagulation and endothelial function. Thus, normal saline may be considered to be a superior form of treatment in terms of coagulation. However it is pertinent to ask whether this response is ultimately beneficial.

### Burn

Two papers analyzed experimental burn models. Hernekamp et al.^[39]^ analyzed the effects of plasma obtained from rats submitted to thermal burn and transferred to healthy unburnt rats. Cinanserin, a specific 5-HT2 receptor-blocking agent was administered to determine whether burn induced systemic edema can be reduced. Intravital microscopy was performed in mesenteric venules to determine systemic edema by FITC-albumin extravasation. Additionally, leukocyte activation (cells/ mm2) was observed. Burn-plasma-transfer results in systemic capillary leakage leading to systemic burn edema. However, intraveneous application of Cinanserin abolished this response. They conclude that this specific 5-HT2 antagonism reduces systemic burn edema and leukocyte activation after plasma transfer. Reduction of capillary leakage may be partially mediated by leukocyte dependent as well as independent mechanisms. The entire subject of vascular permeability in burn injury has been expertly reviewed by Huang et al.^[40]^ covering a large amount of scientific information in many instances published in Chinese periodicals and infrequently cited in the western scientific press. Very detailed data from these publications are reproduced and should be carefully analyzed by readers interested in the theme. 

### Sepsis

This is not a comprehensive examination of published work on sepsis, but exclusively about articles on sepsis accompanying experimental shock procedures. Maybauer et al.^[41]^ used a well established ovine model of septic shock following severe smoke inhalation injury to perform a prospective, randomized, controlled experimental study; the object of this study was to determine the effects of the peroxynitrite decomposition catalyst WW-85 on global hemodynamics and regional microvascular blood flow. The untreated control group developed a hypotensive, hyperdynamic circulation condition and increased plasma levels of nitrate and nitrite. All hemodynamic variables were significantly improved in the treatment group. Cerebral blood flow deteriorated in controls, but was significantly improved in cerebral cortex, cerebellum, pons, medulla oblongata, and thalamus by WW-85. These results provide evidence that WW-85 blocks NO production, thereby improving cardiovascular function and microcirculation. Miranda et al.^[42]^ investigated the effects of heparin on endotoxemia-related microcirculatory changes and compared them to those observed with the use of recombinant human activated protein C. Heparin decreased lipopolysaccharide-induced leukocyte rolling and arteriolar vasoconstriction; survival increased in treated vs. non-treated animals. Administration of heparin plus recombinant human activated protein C was associated with a significant attenuation of lipopolysaccharide-induced capillary perfusion deficits. They claim that heparin yields protective effects on endotoxemic animals' microcirculation. Those benefits were potentiated when heparin was administered in conjunction with recombinant human activated protein C. 

### Small volume hypertonic resuscitation

No review on shock and/or trauma by this writer would be complete without reference to this final theme. It became a theme of intense basic and clinical research in the seventies and eighties as a consequence of research in which this writer played a role and is still present in current reports. A comprehensive review on the subject has been published elsewhere^[43]^, but a number of new reports have appeared. Gong et al.^[44]^ evaluated the effect of 5% hypertonic saline on the interaction of neutrophils and endothelial cells in the blood brain microcirculation. They find that at this unique endothelial barrier neutrophil-endothelial crosstalk is activated in contrast to what occurs in other locations. Granfeldt et al.^[45]^ combined 7.5% NaCl with adenosine, lidocaine and magnesium (Mg^2+)^ and found significant improvements in cardiac index and oxygen delivery vs. conventional 7.5% NaCl. The increase in cardiac index was due to a twofold increase in cerebrovascular accident volume. It is obvious that adenosine and Mg may have been instrumental in this response; the role played by lidocaine requires further investigation. Dubick et al.^[46]^ report that hypertonic acetate resuscitation from aortic cross-clamping offered only minor advantages over hypertonic bicarbonate in terms of blood flow and anti-oxidant status. This observation should be coupled with a previously reported one^[1]^ showing that hypertonic acetate induces significant pulmonary vasoconstriction. It is consequently safe to state that hypertonic acetate is not an adequate resuscitative solution. In the only report cited in this review which is not an animal model, Mola et al.^[47]^ tested the safety and efficacy of hypertonic saline vs isotonic saline in off-pump coronary artery bypass grafting and found that the solution is safe and raised venous pressure return during the critical period of arterial anastomosis. As reported above, Dekker et al.^[38]^ found that treatment of traumatic brain injury plus hemorrhage with hypertonic saline + hydroxyethylstarch is less effective that normal saline treatment. Also as reported above, Marks et al.^[34]^ revisited the theme of hypertonic saline vs. mannitol for the treatment of traumatic brain injury. Taken together, these reports indicate a general shrinkage of interest surrounding small volume hypertonic resuscitation, which may eventually turn out to be useful in different scenarios, as for instance in cardiac surgery. 

## CONCLUSION

This provocative collection of articles describing experimental models of the effects of shock and trauma upon the cardiovascular system indicate that valuable information drawn from experimental models has been developed over the past 5 years concerning endothelial stability, mesenteric lymph, vascular reactivity, traumatic injuries, burn and sepsis. The author is obviously very glad to note that a surviving interest in hypertonic saline still persists.

**Table:** 

**Authors' roles & responsibilities**	
MRS	Analysis/interpretation of data; manuscript writing or critical review of its contents; final manuscript approval
